# Geographic variation in shoot structure in association with fruit size in an evergreen woody species

**DOI:** 10.1093/aobpla/plab023

**Published:** 2021-05-07

**Authors:** Takuma Goto, Noriyuki Osada

**Affiliations:** Laboratory of Plant Conservation Science, Faculty of Agriculture, Meijo University, Nagoya 468-8502, Japan

**Keywords:** *Camellia japonica*, intraspecific variation, leaf area, shoot allometry, stem diameter

## Abstract

The generality of scaling relationships between multiple shoot traits, known as Corner’s rules, has been considered to reflect the biomechanical limits to trees and tree organs among the species of different leaf sizes. Variation in fruit size within species would also be expected to affect shoot structure by changing the mechanical and hydraulic stresses caused by the mass and water requirement of fruits. We investigated the differences in shoot structure and their relationship with fruit size in *Camellia japonica* from 12 sites in a wide geographic range in Japan. This species is known to produce larger fruits with thicker pericarps in more southern populations because warmer climates induce more intensive arms race between the fruit size and the rostrum length of its obligate seed predator. We found that, in association with the change in fruit size, the diameter and mass of 1-year-old stems were negatively associated with latitude, but the total mass and area of 1-year-old leaves did not change with latitude. Consequently, the length of 1-year-old stems and the total mass and area of 1-year-old leaves at a given stem diameter were positively associated with latitude in the allometric relationships. In contrast, the allometric relationships between stem diameter and total mass of the 1-year-old shoot complex (the leaves, stems and fruits that were supported by a 1-year-old stem) did not differ across the trees of different latitudes. Thus, natural selection on fruit size is considered to influence the other traits of Corner’s rules in *C. japonica*, but all of the traits of Corner’s rules do not necessarily change in a similar manner across latitudinal gradients.

## Teaser

*Camellia japonica* is known to produce larger fruits in more southern populations because of intensive arms race between the fruit size and the rostrum length of its obligate seed predator. We found that such geographic changes in fruit size resulted in thicker 1-year-old stems in more southern populations to withstand the loads and/or water requirements of fruits. Such patterns were constrained by the allometric relationships between stem diameter and total mass of the 1-year-old shoot complex (the leaves, stems and fruits that were supported by a 1-year-old stem), which did not differ across the trees of different latitudes.

## Introduction

A shoot is a fundamental unit of growth in woody plants, and the shoot structure has multiple functions and develops under multiple selection pressures (Pearcy *et al.* 2005; Valladares and Niinemets 2007). That is, the shoot is essential to display leaves to receive light and photosynthesize efficiently (Valladares and Pearcy 1998; Falster and Westoby 2003), and is determined by the tradeoffs between mechanical support, conduction and storage of water, carbon and nutrients (Pratt and Jacobsen 2017). Consequently, shoot structure is critically important for the efficient photosynthesis and the growth and survival of shoots and individual trees (Pearcy *et al.* 2005; Valladares and Niinemets 2007).

Interspecific differences in the shoot structure of woody plants have been studied intensively in association with variations in leaf size (Corner 1949; White 1983; Ackerly and Donoghue 1998; Brouat *et al.* 1998), leaf size/number trade-off (Kleiman and Aarssen 2007; Milla 2009) and light interception efficiency (Falster and Westoby 2003; Osada and Hiura 2017). These studies found clear coordination of the shoot structure among species, known as Corner’s rules; the species with thicker stems have fewer and larger leaves (Corner 1949; White 1983; Ackerly and Donoghue 1998; Brouat *et al.* 1998). Notably, such pattern deviates among the species of different habitats; the mass and area of leaves deployed by a stem of given diameter (or cross-sectional area) are smaller for the species of drier regions (Preston and Ackerly 2003; Westoby and Wright 2003; Yang *et al.* 2014) and higher altitudes (Sun *et al.* 2006). The observed pattern of shoot coordination has been explained by the natural selection that favours the avoidance of self-shading and thus captures similar amounts of carbon per unit of crown area, regardless of leaf size (Olson *et al.* 2009, 2018; Messier *et al.* 2017; Smith *et al.* 2017).

Here, we should be careful in considering the coordination of shoot structure when comparing across and within species. Interspecific studies on shoot allometry may inform an understanding of how shoot structure is constrained in trees, due to natural selection (Preston and Ackerly 2003; Westoby and Wright 2003; Yang *et al.* 2014). On the other hand, intraspecific variation in shoot structure and scaling in response to shorter-term environmental gradients, better inform an understanding of tree responses to environmental conditions (Osada *et al.* 2015; Fajardo 2016; Trueba *et al.* 2016; Trejo *et al.* 2018). Thus, interspecific studies provide generality and intraspecific studies provide mechanistic details and plastic responses (Olson and Arroyo-Santos 2015). In this study, we focus on intraspecific differences. In addition, shoot structure might be constrained not only by the size of leaves and stems but also by the size of other appendages such as fruits; the stems should be thicker and/or stiffer to deploy larger fruits to withstand mechanical stress caused by fruit loads and hydraulic stresses caused by water requirements by fruits. Alternatively, although a decrease in leaf mass may compensate for the increase in fruit mass, the reduction in leaf mass might directly reduce the productivity at the shoot level and thus might be disadvantageous for the survival and growth of trees. According to Corner’s rules, the sizes of inflorescences and seeds are expected to be greater for thicker stems (Corner 1949; Midgley and Bond 1989; Cornelissen 1999; Chen *et al.* 2009). Because seed mass tends to be greater for more shade-tolerant species (Hewitt 1998; Walters and Reich 2000), the shoot structure may also be related to shade tolerance. The tree individuals with larger leaves and fewer branches tend to have fewer meristems for deploying fruits, so selection would favour larger fruits than in individuals with smaller leaves and finer branches. Similarly, the selection on fruit size would result in similar shoot trait combinations, such as larger leaves, thick and fewer branches. To our knowledge, these hypotheses related to variation in fruit size within species have not been focussed on in the studies of shoot structure.

**Figure 1.**
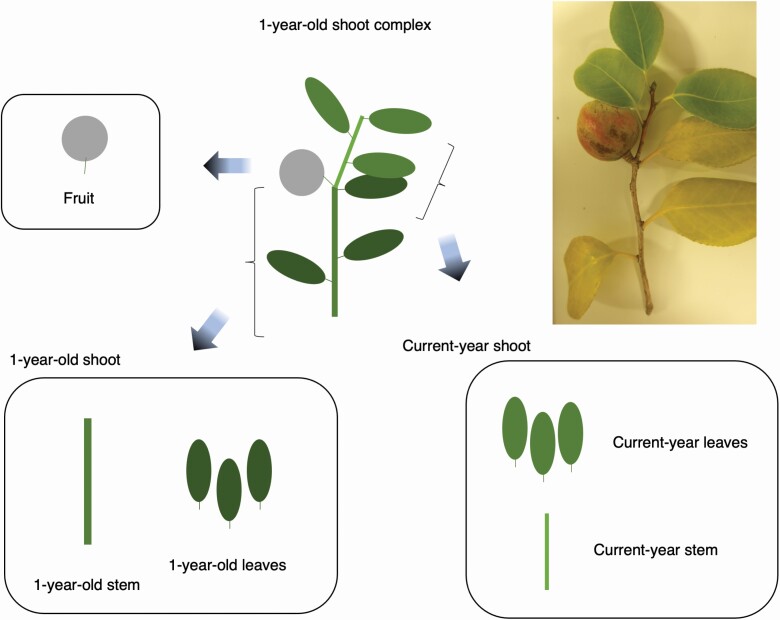
A schema of a fruiting 1-year-old shoot complex of *Camellia japonica*.

*Camellia japonica* (Theaceae) is a shade-tolerant evergreen broad-leaved species that shows a large geographic variation in fruit size because of the predator–prey interactions (Okamoto 1988; Toju and Sota 2006; Toju 2011). The arms race between the fruit size of *C. japonica* and the rostrum length of its obligate seed predator, *Curculio camelliae* (Coleoptera: Curculionidae) is strengthened in southern populations due to warm climates, and consequently, the *Camellia* trees of more southern populations have larger fruits with thicker pericarps (Toju and Sota 2006; Toju *et al.* 2011). Such a latitudinal gradient in fruit size provides an opportunity to test the hypothesis of whether or not stem allometry scales as a function of fruit size gradient in this species. In addition, previous studies indicated that fruit diameter and pericarp thickness are negatively correlated with latitude in this species (Toju and Sota 2006; Toju *et al.* 2011). We, therefore, focussed on the relationship between fruit diameter and fruit mass, because the decrease in fruit tissue density might compensate for the increase in fruit diameter, and might result in similar fruit mass regardless of fruit size variation. We expected that shoot structure is constrained by the loads of fruit mass, and thus the relationships among mass, diameter and tissue density of fruits need to be considered for evaluating the fruit effects on shoot structure. Moreover, the fruits are deployed by 1-year-old stems in this species, and the 1-year-old stems are constrained to withstand not only the loads and water requirements of fruits but also by those of current-year shoots (Fig. 1). We thus focussed not only on fruits but also on current-year shoots that were deployed by 1-year-old shoots.

**Figure 2.**
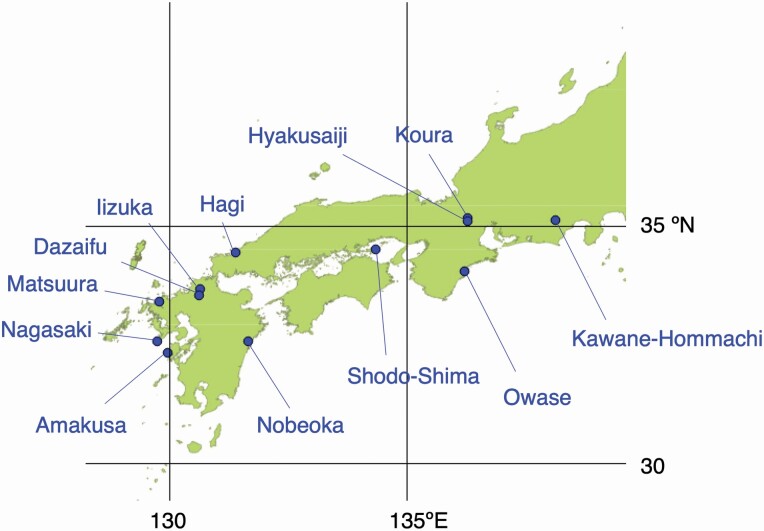
Locations of 12 sampling sites of *Camellia japonica* in southern Japan.

We investigated the differences in shoot structure and their relationship with fruit size in *C. japonica* in southern Japan, with a latitudinal range from 32.3 to 35.4°N (Fig. 2 and Table 1). Fruit-bearing 1-year-old shoot complexes (current-year and 1-year-old stems, leaves and fruits that were supported by a given 1-year-old stem) were collected from 12 sites in a wide geographic range (Figs 1 and 2). We expected that natural selection that acts on fruit size influences the other traits of Corner’s rules, and consequently shoot structure changes with latitude and fruit size. We particularly predicted the following: (i) In association with the change in fruit size, the diameter and mass of 1-year-old stems are negatively associated with latitude, but the total mass and area of 1-year-old leaves do not change with latitude. (ii) The total mass of the 1-year-old shoot complex is negatively associated with latitude, but the total leaf area of the 1-year-old shoot complex does not change with latitude. (iii) Stem diameter increases not only with total leaf area and total leaf mass of the 1-year-old shoots but also with those of current-year shoots and the mass of fruits. (iv) Latitudinal changes in shoot allometry result in a similar allometric relationship between stem diameter and total leaf area and between stem diameter and total mass of the 1-year-old shoot complex across the trees of different latitudes.

**Table 1.** Information and mean annual temperature of the study sites.

Site	Latitude (°N)	Longitude (°E)	Altitude (m)	Mean temperature (°C)
Koura	35.18	136.28	300	13.57
Kawane-Hommachi	35.14	138.14	370	13.45
Hyakusaiji	35.13	136.28	300	13.57
Shodo-Shima	34.52	134.35	30	15.55
Owase	34.06	136.23	80	15.82
Iizuka	33.68	130.65	63	15.61
Dazaifu	33.54	130.61	220	14.74
Hagi	33.46	131.39	12	15.47
Matsuura	33.41	129.79	26	16.33
Nagasaki	32.58	129.74	186	16.37
Nobeoka	32.58	131.66	10	16.68
Amakusa	32.35	129.97	40	16.40

## Materials and Methods

### Study species

*Camellia japonica* is a shade-tolerant evergreen woody species and distributes in warm-temperate forests in Japan and the southern and western coasts of the Korean Peninsula (Aiba and Kohyama 1996; Ueno *et al.* 2000; Kubota *et al.* 2014). Maximum height is about 13 m in this species and abundant in a shaded forest understory (Aiba and Kohyama 1996, 1997), and the third dominant species in stem number in a 4-ha plot in Tsushima, Japan (Ueno *et al.* 2000). This species produces flowers from winter to spring and is effectively pollinated by birds such as *Zosterops japonica* (Osada *et al.* 2003; Kunitake *et al.* 2004). The fruits gradually develop from spring to summer (Abe *et al.* 2006). Because new leaves emerge from leafy buds in late spring (Osada 2017), the mature fruits are deployed by 1-year-old shoots (Fig. 1). Each fruit usually contains 3–7 seeds surrounded by a thick pericarp (Toju and Sota 2006).

### Shoot sampling

Twelve sites with a wide geographic range were chosen as the study sites (Fig. 2). The fruit size of *C. japonica* is known to vary greatly in this geographic range (Okamoto 1988; Toju and Sota 2006; Toju 2011). The mean annual temperature of study sites was calculated from data at the weather stations of the Japan Meteorological Agency near the sites and altitudinal differences between the sites and weather stations with the lapse rate of 0.55 °C per 100 m (Table 1). The mean annual temperature was negatively correlated with latitude (*r* = −0.86, *P* = 0.0003).

Four to five individual trees of approximately 5 m height and 3–5 cm in trunk diameter at breast height were selected at each site, and one 1-year-old shoot that supported fruits was harvested from the middle to top of the crown of each tree in August and September 2018. The harvested shoots were exposed to high light in all trees. The fruits matured but had not dehisced at the time of collection at all sites.

Immediately after collection, each shoot was divided into one of the following categories: 1-year-old stem, 1-year-old leaves, current-year stems, current-year leaves and fruits (Fig. 1). Current-year leaves and stems can be distinguished from 1-year-old leaves and stems because of the brighter colours in the former and clear scars of bud scales. Also, fruits are deployed by 1-year-old stems and thus the position of fruits can be used for identifying 1-year-old stems. The length of 1-year-old stems and the number of standing leaves were measured, and the diameter of 1-year-old stems was measured at 10 % of the length from the base of the 1-year-old stems. Each leaf was scanned to a computer, and the leaf area was measured using Image J software (NIH, Oklahoma, USA). The diameter and fresh mass were measured for each fruit, and the fruit volume was measured using the water displacement method. Fruit density was calculated as the fresh mass divided by volume. The fruits, stems, and leaves were then oven-dried at 60 °C for more than one week, and the dry mass was measured. The volume of 1-year-old stems was calculated by assuming the cylinder with measured diameter and length, and the wood density of 1-year-old stems was calculated as the dry mass divided by volume.

### Statistics

Because the latitude was negatively correlated with mean temperature and the fruit size was expected to change with latitude (Toju and Sota 2006; Toju *et al.* 2011), the relationships between latitude and shoot traits were investigated using generalized linear models (GLMs) in the statistical software R (R Development Core Team 2018). Gamma distribution was applied for the shoot traits, i.e. the fresh mass, diameter, volume and tissue density of fruits, the dry mass, length and diameter of 1-year-old stems, the total dry mass, total area and leaf mass per area (LMA) of 1-year-old leaves and total dry mass and total leaf area of 1-year-old shoot complex, with the link function of the log, except for the number of leaves per 1-year-old shoot, in which a Poisson distribution was applied. Note that the Gamma distribution with the link function of log is effective in describing larger variation in the traits of larger value.

The mass of the current-year shoots deployed by a given 1-year-old shoot may also affect the structures of 1-year-old shoots because of their loads. The latitude, the existence of current-year shoots, and their interaction term were, therefore, parameterized in the GLM. In addition, the latitudinal effect on shoot allometry was examined by categorizing the latitude of study sites to the four classes of a 1° range for simplicity. It would be inappropriate to minimize the sums of squares in only the y-dimension for these relationships because both variables exhibit variation due to measurement errors. Thus, the allometric relationships between pairs of shoot traits were investigated for the four classes of latitude using a standard major axis analysis in the sma function in the smatr package (Warton *et al.* 2012) with the statistical software R. The differences in slopes and intercepts in the allometric relationship between the four latitude classes were tested using likelihood methods (Falster *et al.* 2006).

## Results

### Latitudinal changes in shoot traits

**Figure 3.**
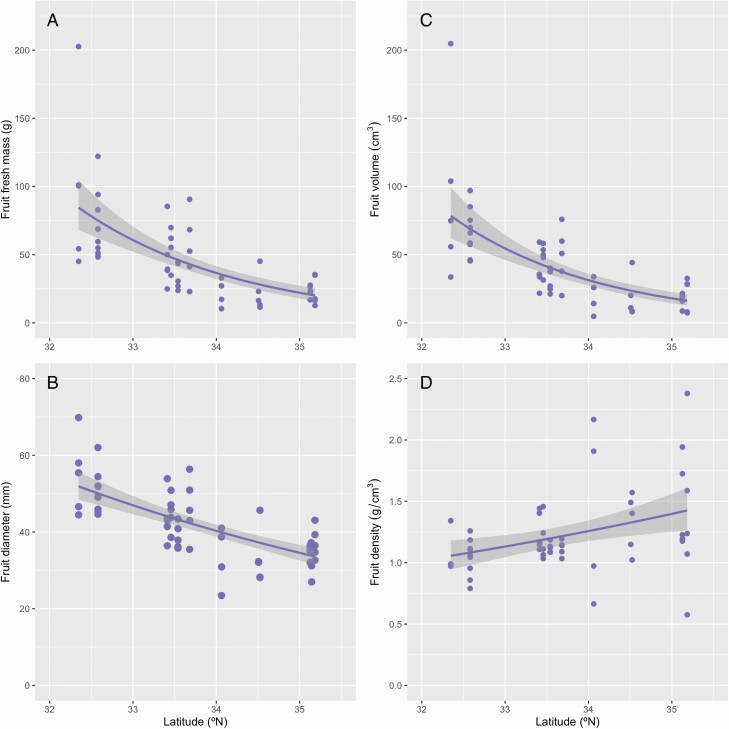
Relationships between fruit traits (A, fruit fresh mass; B, fruit diameter; C, fruit volume; D, fruit density) and latitude of *Camellia japonica*. The results of GLM and 95 % confidence intervals are shown as lines and gray zones, respectively. See Table 1 for statistical results.

Fresh mass, volume and diameter of individual fruits were negatively associated with latitude (Fig. 3A–C and Table 2; see Supporting Information—File S1). In contrast, fruit tissue density was positively associated with latitude (Fig. 3D), but the difference in mean density between sites was small (1.2-fold difference in mean density between the northernmost and the southernmost sites). Consequently, the latitudinal difference in the fruit fresh mass (4.3-fold) was much greater than that of the fruit diameter (1.5-fold; Fig. 3A and B).

**Table 2.** Results of GLMs for effects of latitude on fruit traits.

Fruit traits	*N*		Estimate	SE	*t*	*P*
Fresh mass (g)	53	Intercept	20.80	2.26	9.22	<0.0001
		Latitude	−0.51	0.07	−7.56	<0.0001
Diameter (mm)	59	Intercept	8.90	0.67	13.26	<0.0001
		Latitude	−0.15	0.02	−7.72	<0.0001
Volume (cm^3^)	53	Intercept	22.33	2.47	9.03	<0.0001
		Latitude	−0.56	0.07	−7.57	<0.0001
Tissue density (g/cm^3^)	53	Intercept	−3.39	1.18	−2.88	0.0059
		Latitude	0.11	0.03	3.04	0.0037

**Figure 4.**
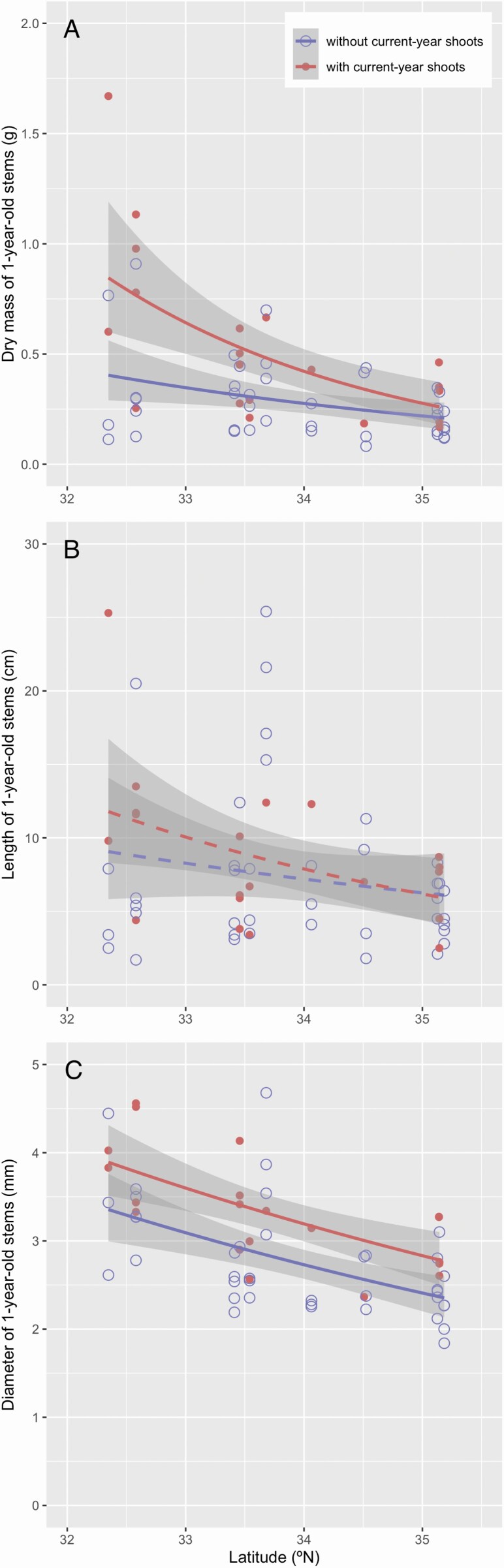
Relationships between traits of 1-year-old stems (A, dry mass of 1-year-old stems; B, length of 1-year-old stems; C, diameter of 1-year-old stems) and latitude of *Camellia japonica*. The shoots with and without current-year shoots are shown by dots and circles, respectively. The regression lines of GLM and 95 % confidence intervals are shown as lines and gray zones, respectively (solid lines, *P* < 0.05; broken lines, *P* > 0.05). See Table 2 for statistical results.

**Figure 5.**
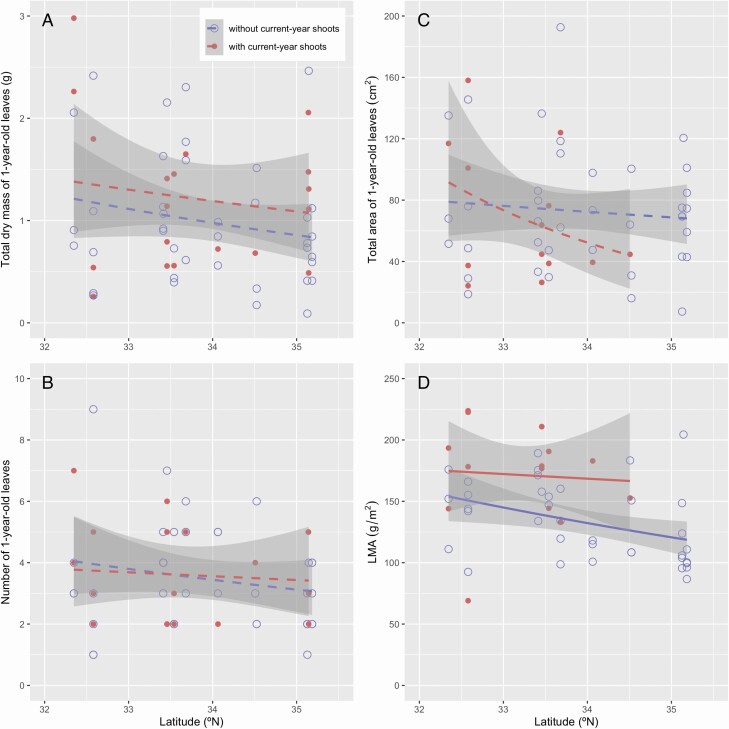
Relationships between traits of 1-year-old leaves (A, total dry mass of 1-year-old leaves; B, number of 1-year-old leaves; C, total area of 1-year-old leaves; D, LMA) and latitude of *Camellia japonica*. The shoots with and without current-year shoots are shown by dots and circles, respectively. The regression lines of GLM and 95 % confidence intervals are shown as lines and gray zones, respectively (solid lines, *P* < 0.05; broken lines, *P* > 0.05). See Table 2 for statistical results.

The dry mass and diameter of 1-year-old stems were negatively associated with latitude, whereas the length of 1-year-old stems was not associated with latitude (Fig. 4A–C and Table 3). Wood density of 1-year-old stems was 0.78 ± 0.05 g cm^−3^ (mean ± SE), and was not associated with latitude (GLM; *P* = 0.084). Although the 1-year-old stems that deployed current-year shoots seemed to be thicker with more dry mass than those that did not produce current-year shoots, these differences were insignificant (Fig. 4A and C and Table 3). Note that the number of current-year shoots per 1-year-old shoot was mostly zero or one, with a mean value of >1 at only one site (2.25 in Nobeoka). As a consequence, the number of current-year shoots was not associated with latitude (*P* = 0.076). The total mass, total area and the number of 1-year-old leaves per 1-year-old shoot were variable for the trees of similar latitudes, and were related neither to latitude nor to the existence of current-year shoots (Fig. 5A–C and Table 3). In contrast, LMA was negatively associated with latitude but was not related to the existence of current-year shoots, although the variance was large within the site (Fig. 5D and Table 3).

**Table 3.** Results of GLMs for effects of latitude, the existence of current-year shoots and their interaction on the shoot traits.

Traits		*N*		Estimate	SE	*t*	*P*
1-year-old stem	Dry mass (g)	59	Intercept	6.54	2.95	2.22	0.031
			Latitude	−0.23	0.09	−2.65	0.011
			Current-year shoot	6.91	4.94	1.40	0.17
			Interaction	−0.19	0.15	−1.30	0.20
	Length (cm)	59	Intercept	6.72	3.72	1.80	0.077
			Latitude	−0.14	0.11	−1.27	0.21
			Current-year shoot	3.62	6.24	0.58	0.56
			Interaction	−0.10	0.18	−0.56	0.58
	Diameter (mm)	59	Intercept	5.25	0.99	5.33	<0.0001
			Latitude	−0.12	0.03	−4.30	<0.0001
			Current-year shoot	−0.01	1.65	0.00	1.00
			Interaction	0.00	0.05	0.10	0.92
1-year-old leaves	Total dry mass (g)	59	Intercept	4.42	3.49	1.27	0.21
			Latitude	−0.13	0.10	−1.27	0.21
			Current-year shoot	1.19	5.84	0.20	0.84
			Interaction	−0.03	0.17	−0.16	0.87
	Total area (cm^2^)	52	Intercept	6.05	3.10	1.95	0.057
			Latitude	−0.05	0.09	−0.57	0.57
			Current-year shoot	14.43	8.05	1.79	0.079
			Interaction	−0.44	0.24	−1.80	0.078
	Number	59	Intercept	4.56	2.98	1.53	0.13
			Latitude	−0.10	0.09	−1.11	0.27
			Current-year shoot	−2.09	4.97	−0.42	0.67
			Interaction	0.06	0.15	0.42	0.67
	LMA (g/m^2^)	52	Intercept	8.48	1.47	5.79	<0.0001
			Latitude	−0.11	0.04	−2.44	0.019
			Current-year shoot	−2.60	3.81	−0.68	0.50
			Interaction	0.08	0.11	0.73	0.47
1-year-old shoot complex	Total dry mass (g)	53	Intercept	16.65	2.10	7.94	<0.0001
			Latitude	−0.41	0.06	−6.69	<0.0001
			Current-year shoot	3.02	5.34	0.57	0.58
			Interaction	−0.09	0.16	−0.56	0.58
	Total leaf area (cm^2^)	51	Intercept	7.03	2.90	2.42	0.019
			Latitude	−0.08	0.09	−0.95	0.35
			Current-year shoot	16.60	7.40	2.24	0.030
			Interaction	−0.48	0.22	−2.16	0.036

**Figure 6.**
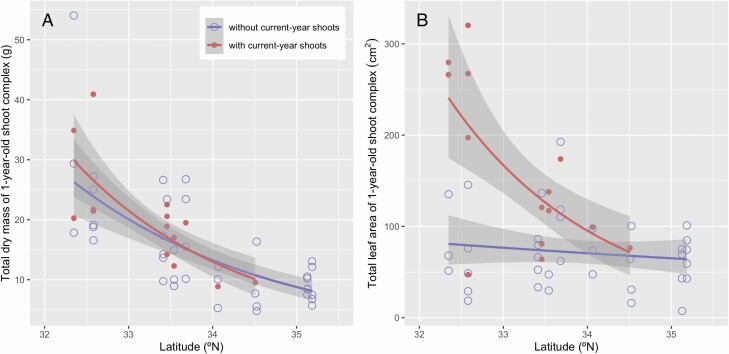
Relationships between traits of 1-year-old shoot complexes (A, total mass of 1-year-old shoot complex; B, total leaf area of 1-year-old shoot complex) and latitude of *Camellia japonica*. The shoots with and without current-year shoots are shown by dots and circles, respectively. The regression lines of GLM and 95 % confidence intervals are shown as lines and gray zones, respectively. See Table 2 for statistical results.

The total dry mass of the 1-year-old shoot complex was larger for the trees of more southern sites, and the existence of current-year shoots did not affect this pattern (Fig. 6A and Table 3). The total leaf area of the 1-year-old shoot complex was larger for the trees of more southern sites, and was greater for the 1-year-old shoots that deployed current-year shoots than those that did not deploy current-year shoots. This was particularly exhibited at the southern sites, resulting in the interaction of latitude and the existence of current-year shoots (Fig. 6B and Table 3).

### Latitudinal changes in allometric relationships

**Figure 7.**
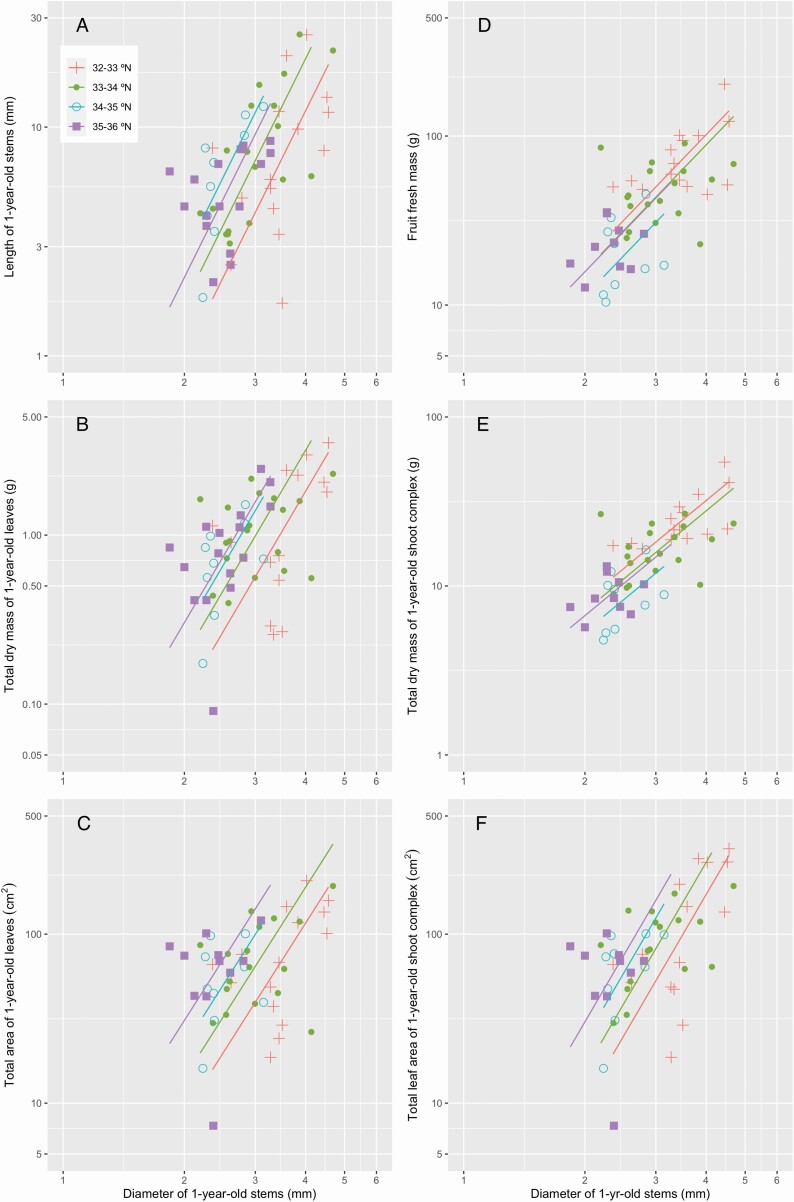
Allometric relationships between diameter of 1-year-old stems and the traits of 1-year-old shoots, fruits, and 1-year-old shoot complexes (A, length of 1-year-old stems; B, total dry mass of 1-year-old leaves; C, total area of 1-year-old leaves; D, fruit fresh mass; E, total dry mass of 1-year-old shoot complex; F, total leaf area of 1-year-old shoot complex). Standard major axis regressions are shown for the trees of different latitude classes. See Table 3 for statistical results.

The slopes of the allometric relationships between the diameter of 1-year-old stems and studied shoot traits did not differ among the four latitude classes for all of the combinations considered (Fig. 7A–F and Table 4). Intercepts of the allometric relationships differed in a constant direction, and the length of 1-year-old stems, total dry mass and total area of 1-year-old leaves, and total leaf area of 1-year-old shoot complex were smaller for the trees of more southern sites (Fig. 7A–C and F and Table 4). Thus, the 1-year-old stems of a given diameter were shorter and supported less mass and area of leaves for the trees of more southern sites. Conversely, the intercepts did not differ significantly in the relationship between the diameter of 1-year-old stems and the fresh mass of fruits that were supported by the stems, and between the diameter of 1-year-old stems and the total dry mass of the 1-year-old shoot complex (Fig. 7D and E and Table 4). These results suggest that the 1-year-old stems of a given diameter supported similar fruit mass and total dry mass of 1-year-old- shoot complex regardless of latitude class.

**Table 4.** Results of standard major axis regression in the allometric relationships between the diameter of 1-year-old stems and shoot traits. Estimates of the allometric slopes with 95 % confidence intervals (CIs) are shown with statistical results. The likelihood ratio was used to investigate the difference of slopes among the four latitude classes, and if the slopes were not significantly different (*P* > 0.05), the difference of intercepts was investigated by the Wald statistic (Falster *et al.* 2006).

	Slope						Intercept		
Shoot traits	Estimate	Lower CI	Upper CI	Likelihood ratio	df	*P*	Wald statistic	df	*P*
Length of 1-year-old stems	3.56	2.78	4.45	2.92	3	0.40	13.24	3	0.0042
Total dry mass of 1-year-old leaves	4.06	3.07	5.23	4.05	3	0.26	9.39	3	0.025
Total area of 1-year-old leaves	3.75	2.76	4.98	2.42	3	0.49	11.26	3	0.010
Fresh mass of fruits	2.48	1.83	3.26	2.17	3	0.54	3.97	3	0.26
Total dry mass of 1-year-old shoot complex	1.97	1.49	2.55	2.47	3	0.48	5.68	3	0.13
Total leaf area of 1-year-old shoot complex	4.09	3.01	5.36	5.19	3	0.16	8.26	3	0.041

## Discussion

Corner’s rules describe a global spectrum from the species with large leaves and thick and sparsely branched stems of low wood density to those with small leaves and thin and highly branched stems of high wood density, and are found across species in a number of studies (Preston and Ackerly 2003; Westoby and Wright 2003; Sun *et al.* 2006; Olson *et al.* 2009, 2018; Yang *et al.* 2014). In *C. japonica*, the negative relationship between fruit size and latitude has been attributed to the intensification of co-evolutionary arms races between the fruit size (pericarp thickness) and the rostrum length of specific weevils in the regions of warm climate (Toju and Sota 2006; Toju *et al.* 2011). We found that the structure of fruiting shoots differed between *C. japonica* trees at different latitudes, and this difference was related to the latitudinal gradient of fruit size (Figs 3–7). Thus, natural selection on fruit size is considered to influence the other traits of Corner’s rules in this species.

As a consequence, our predictions were partly supported: (i) In association with the change in fruit size, the diameter and mass of 1-year-old stems were positively associated, but the total mass and area of 1-year-old leaves were not associated with latitude. (ii) The total mass and total leaf area of the 1-year-old shoot complex were negatively associated with latitude. (iii) The length of 1-year-old stems, total mass and area of 1-year-old leaves at a given stem diameter were positively associated with latitude in the allometric relationships. This resulted in a similar allometric relationship between the stem diameter and total mass of the 1-year-old shoot complex across the trees of different latitude classes but not between the stem diameter and total leaf area of the 1-year-old shoot complex.

The mechanical basis of Corner’s rules have been considered as follows: if similar crown areas fix similar amounts of carbon regardless of leaf size, then large-leaved species, with their distantly spaced leaves, require higher stem growth rates, lower stem tissue densities and stiffnesses and, therefore, thicker twigs (Olson *et al.* 2018). As a consequence, leaf size, the thickness, density and spacing of branches and growth rate are expected to be correlated with one another across species. Within species, however, some of these traits might be more plastic than others. For example, wood density is determined by the anatomical characteristics of the species and is phylogenetically constrained (Zanne *et al.* 2010), and might be less plastic than other traits of Corner’s rules. In accordance with this, the increase in fruit size was associated with that of stem diameter and mass, but not that of stem density in *C. japonica*. Similarly, allometric relationship between the stem diameter and total mass of the 1-year-old shoot complex did not differ. However, allometric relationship between stem diameter and total leaf area of the 1-year-old shoot complex differed across the trees of different latitude classes (Fig. 7). This suggests mechanical and/or hydraulic stress of fruits affected the stem structure but did not affect the leaf area of *C. japonica*. Thus, all of the traits of Corner’s rules do not necessarily show similar plastic responses within species.

In addition to fruit size, the existence of current-year shoots might affect the structure and biomass of 1-year-old shoots by increasing the mechanical and hydraulic stresses. In this study, the diameter was greater for the 1-year-old stems of lower latitudes, but the total dry mass and the total area of the 1-year-old leaves were not associated with latitude (Fig. 4C and 5A and C). The existence of current-year shoots seemed to influence the structure of 1-year-old stems but this potential effect was not statistically detected, potentially due to the large variance within the site. These results suggest that the difference in the structure and biomass of the 1-year-old stems was not caused by the 1-year-old leaves on the shoots. Also, the total dry mass of the 1-year-old shoot complex was negatively associated with latitude, but the existence of current-year shoots did not bias the results (Fig. 6A). This is primarily because very large fruits of this species constitute most of the total dry mass of the 1-year-old shoot complex (88.3 ± 6.7 % in mean ± SD). In contrast, the interaction between latitude and the existence of current-year shoots was detected for the total leaf area of the 1-year-old shoot complex (Fig. 6B), because of the production of current-year shoots and thus an increase in the leaf area for the trees at lower latitudes. Therefore, the effect of total mass on stem diameter, but not that of total leaf area, converged in the structural relationship of 1-year-old stems in *C. japonica* at the geographic scale.

Allometric relationships between various shoot traits changed with latitude, and stem length, total dry mass, area of the 1-year old leaves, and total leaf area of the 1-year-old shoot complex at a 1-year-old stem of a given diameter were negatively associated with latitude class. Previous studies have focussed on current-year shoots or unbranched twigs, but similar relationships between stem diameter and total leaf area and that between stem diameter and total leaf mass were found among species in response to drought (Preston and Ackerly 2003; Westoby and Wright 2003; Yang *et al.* 2014) and increasing altitude (Sun *et al.* 2006). Also, similar relationships were found within species in response to drought (Yang *et al.* 2014) and increasing tree height (Osada *et al.* 2002, 2004, 2014; Osada 2006, 2011) (but see Miyata *et al.* 2011). Latitudinal changes in temperature may have similar effects to drought and tree height in *C. japonica*.

Although we did not investigate the shoots without fruits, differences in the structure between the shoots with and without fruits are noteworthy because the fruit mass of *C. japonica* is greater than that of most other native tree species in Japan. In particular, mechanical properties should be studied for the fruiting and non-fruiting shoots in addition to the structural traits considered in this study, because the trade-off between the Young’s modulus (stem material stiffness) and the second moment of area (stem transectional size and geometric property) is also an important feature of Corner’s rules (Olson *et al.* 2018). Fruiting stems of *C. japonica* often droop, implying that the stem diameter would not compensate for loads of fruits against buckling (N. Osada, pers. obs.). This suggests that the diameter of fruiting stems would be important to conduct water and nutrients and/or to prevent rupture but not buckling. It is also important to investigate whether the fruiting shoots similarly contribute to future growth in crowns in comparison to non-fruiting shoots because the trade-off between growth and reproduction may result in the differentiation of fruiting and non-fruiting shoots in the structure and biomass allocation patterns (Obeso 1997; Henriksson and Ruohomäki 2000; Hasegawa and Takeda 2001; Kawamura and Takeda 2006).

Overall, our results strongly suggest that shoot allometry is constrained by fruits in *C. japonica*, and evolutionary changes in fruit size resulted in thicker 1-year-old stems to withstand the loads and/or water requirements of fruits. Effects of fruit size on shoot structure and allometry within species are important to understand how the Corner’s rules are physiologically regulated and restricted both within and among species.

## Supporting Information

The following additional information is available in the online version of this article—

**File S1.** Data used in the study.

plab023_suppl_Supplementary_Materials

## Notes

**Form & Function. Chief Editor:** Kate McCulloh

## References

[CIT0001] Abe H , Matsuki R, Ueno S, Nashimoto M, Hasegawa M. 2006. Dispersal of *Camellia japonica* seeds by *Apodemus speciosus* revealed by maternity analysis of plants and behavioral observation of animal vectors. Ecological Research 21:732–740.

[CIT0002] Ackerly DD , Donoghue MJ. 1998. Leaf size, sapling allometry, and Corner’s rules: phylogeny and correlated evolution in maples (*Acer*). The American Naturalist 152:767–791. 10.1086/286208 18811427

[CIT0003] Aiba S-I , Kohyama T. 1996. Tree species stratification in relation to allometry and demography in a warm-temperate rain forest. Journal of Ecology 84:207–218.

[CIT0004] Aiba S-I , Kohyama T. 1997. Crown architecture and life-history traits of 14 tree species in a warm-temperate rain forest: significance of spatial heterogeneity. Journal of Ecology 85:611–624.

[CIT0005] Brouat C , Givernau M, Amsellem L, McKey D. 1998. Corner’s rules revisited: ontogenetic and interspecific patterns in leaf-stem allometry. The New Phytologist 139:459–470.

[CIT0006] Chen H , Niklas KJ, Yang D, Sun S. 2009. The effect of twig architecture and seed number on seed size variation in subtropical woody species. The New Phytologist 183:1212–1221. 19496950 10.1111/j.1469-8137.2009.02878.x

[CIT0007] Cornelissen JHC . 1999. A triangular relationship between leaf size and seed size among woody species: allometry, ontogeny, ecology and taxonomy. Oecologia 118:248–255. 28307701 10.1007/s004420050725

[CIT0008] Corner EJH . 1949. The durian theory or the origin of the modern tree. Annals of Botany 13:367–414.

[CIT0009] Fajardo A . 2016. Are trait-scaling relationships invariant across contrasting elevations in the widely distributed treeline species *Nothofagus pumilio*? American Journal of Botany 103:821–829. 27208350 10.3732/ajb.1500439

[CIT0010] Falster DS , Warton DI, Wright IJ. 2006. SMATR: standardised major axis tests & routines. http://www.bio.mq.edu.au/ecology/SMATR.

[CIT0011] Falster DS , Westoby M. 2003. Leaf size and angle vary widely across species: what consequences for light interception? New Phytologist 158:509–525. 10.1046/j.1469-8137.2003.00765.x 36056508

[CIT0012] Hasegawa S , Takeda H. 2001. Functional specialization of current shoots as a reproductive strategy in Japanese alder (*Alnus hirsuta* var. *sibirica*). Canadian Journal of Botany 79:38–48.

[CIT0013] Henriksson J , Ruohomäki K. 2000. Assessing costs of reproduction in mountain birch: the importance of considering the modular level. Annals of Botany 86:503–510.

[CIT0014] Hewitt N . 1998. Seed size and shade-tolerance: a comparative analysis of North American temperate trees. Oecologia 114:432–440. 28307788 10.1007/s004420050467

[CIT0015] Kawamura K , Takeda H. 2006. Cost and probability of flowering at the shoot level in relation to variability in shoot size within the crown of *Vaccinium hirtum* (Ericaceae). The New Phytologist 171:69–80. 16771983 10.1111/j.1469-8137.2006.01737.x

[CIT0016] Kleiman D , Aarssen LW. 2007. The leaf size/number trade-off in trees. Journal of Ecology 95:376–382.

[CIT0017] Kubota Y , Hirao T, Fujii Sj, Shiono T, Kusumoto B. 2014. Beta diversity of woody plants in the Japanese archipelago: the roles of geohistorical and ecological processes. Journal of Biogeography 41:1267–1276.

[CIT0018] Kunitake YK , Hasegawa M, Miyashita T, Higuchi H. 2004. Role of a seasonally specialist bird *Zosterops japonica* on pollen transfer and reproductive success of *Camellia japonica* in a temperate area. Plant Species Biology 19:197–201.

[CIT0019] Messier J , Lechowicz MJ, McGill BJ, Violle C, Enquist BJ. 2017. Interspecific integration of trait dimensions at local scales: the plant phenotype as an integrated network. Journal of Ecology 105:1775–1790.

[CIT0020] Midgley J , Bond W. 1989. Leaf size and inflorescence size may be allometrically related traits. Oecologia 78:427–429. 28312592 10.1007/BF00379120

[CIT0021] Milla R . 2009. The leafing intensity premium hypothesis tested across clades, growth forms and altitudes. Journal of Ecology 97:972–983.

[CIT0022] Miyata R , Kubo T, Nabeshima E, Kohyama TS. 2011. Common allometric response of open-grown leader shoots to tree height in co-occurring deciduous broadleaved trees. Annals of Botany 108:1279–1286. 21914698 10.1093/aob/mcr228 PMC3197456

[CIT0023] Obeso JR . 1997. Costs of reproduction in *Ilex aquifolium*: effects at tree, branch and leaf levels. Journal of Ecology 85:159–166.

[CIT0024] Okamoto M . 1988. Interactions between *Camellia japonica* and its seed predator *Curculio camelliae*. II. Stability and dynamics in *Camellia*-*Curculio* interaction. Plant Species Biology 3:99–108.

[CIT0025] Olson ME , Aguirre-Hernández R, Rosell JA. 2009. Universal foliage-stem scaling across environments and species in dicot trees: plasticity, biomechanics and Corner’s rules. Ecology Letters 12:210–219. 19141123 10.1111/j.1461-0248.2008.01275.x

[CIT0026] Olson ME , Arroyo-Santos A. 2015. How to study adaptation (and why to do it that way). The Quarterly Review of Biology 90:167–191. 26285354 10.1086/681438

[CIT0027] Olson ME , Rosell JA, Zamora Muñoz S, Castorena M. 2018. Carbon limitation, stem growth rate and the biomechanical cause of Corner’s rules. Annals of Botany 122:583–592. 29889257 10.1093/aob/mcy089 PMC6153482

[CIT0028] Osada N . 2006. Crown development in a pioneer tree, *Rhus trichocarpa*, in relation to the structure and growth of individual branches. The New Phytologist 172:667–678. 17096793 10.1111/j.1469-8137.2006.01857.x

[CIT0029] Osada N . 2011. Height-dependent changes in shoot structure and tree allometry in relation to maximum height in four deciduous tree species. Functional Ecology 25:777–786.

[CIT0030] Osada N . 2017. Relationships between the timing of budburst, plant traits, and distribution of 24 coexisting woody species in a warm-temperate forest in Japan. American Journal of Botany 104:550–558. 28424203 10.3732/ajb.1600444

[CIT0031] Osada N , Hiura T. 2017. How is light interception efficiency related to shoot structure in tall canopy species? Oecologia 185:29–41. 28801737 10.1007/s00442-017-3926-0

[CIT0032] Osada N , Nabeshima E, Hiura T. 2015. Geographic variation in shoot traits and branching intensity in relation to leaf size in *Fagus crenata*: a common garden experiment. American Journal of Botany 102:878–887. 26101414 10.3732/ajb.1400559

[CIT0033] Osada N , Okabe Y, Hayashi D, Katsuyama T, Tokuchi N. 2014. Differences between height- and light-dependent changes in shoot traits in five deciduous tree species. Oecologia 174:1–12. 23928890 10.1007/s00442-013-2744-2

[CIT0034] Osada N , Sugiura S, Kawamura K, Cho M, Takeda H. 2003. Community-level flowering phenology and fruit set: comparative study of 25 woody species in a secondary forest in Japan. Ecological Research 18:711–723.

[CIT0035] Osada N , Takeda H, Furukawa A, Awang M. 2002. Changes in shoot allometry with increasing tree height in a tropical canopy species, *Elateriospermum tapos*. Tree Physiology 22:625–632. 12069918 10.1093/treephys/22.9.625

[CIT0036] Osada N , Tateno R, Mori A, Takeda H. 2004. Changes in crown development patterns and current-year shoot structure with light environment and tree height in *Fagus crenata* (Fagaceae). American Journal of Botany 91:1981–1989. 21652346 10.3732/ajb.91.12.1981

[CIT0037] Pearcy RW , Muraoka H, Valladares F. 2005. Crown architecture in sun and shade environments: assessing function and trade-offs with a three-dimensional simulation model. The New Phytologist 166:791–800. 15869642 10.1111/j.1469-8137.2005.01328.x

[CIT0038] Pratt RB , Jacobsen AL. 2017. Conflicting demands on angiosperm xylem: tradeoffs among storage, transport and biomechanics. Plant, Cell & Environment 40:897–913. 10.1111/pce.12862 27861981

[CIT0039] Preston KA , Ackerly DD. 2003. Hydraulic architecture and the evolution of shoot allometry in contrasting climates. American Journal of Botany 90:1502–1512. 21659103 10.3732/ajb.90.10.1502

[CIT0040] R Development Core Team . 2018. R: a language and environment for statistical computing. Version 3.5.1. Vienna, Austria: The R Foundation for Statistical Computing.

[CIT0041] Smith DD , Sperry JS, Adler FR. 2017. Convergence in leaf size versus twig leaf area scaling: do plants optimize leaf area partitioning? Annals of Botany 119:447–456. 28028019 10.1093/aob/mcw231 PMC7296615

[CIT0042] Sun S , Jin D, Shi P. 2006. The leaf size-twig size spectrum of temperate woody species along an altitudinal gradient: an invariant allometric scaling relationship. Annals of Botany 97:97–107. 16254019 10.1093/aob/mcj004 PMC2803375

[CIT0043] Toju H . 2011. Weevils and camellias in a Darwin’s race: model system for the study of eco-evolutionary interactions between species. Ecological Research 26:239–251.

[CIT0044] Toju H , Abe H, Ueno S, Miyazawa Y, Taniguchi F, Sota T, Yahara T. 2011. Climatic gradients of arms race coevolution. The American Naturalist 177:562–573. 10.1086/659624 21508604

[CIT0045] Toju H , Sota T. 2006. Imbalance of predator and prey armament: geographic clines in phenotypic interface and natural selection. The American Naturalist 167:105–117. 10.1086/498277 16475103

[CIT0046] Trejo L , Rosell JA, Olson ME. 2018. Nearly 200 years of sustained selection have not overcome the leaf area-stem size relationship in the poinsettia. Evolutionary Applications 11:1401–1411. 30151048 10.1111/eva.12634 PMC6099819

[CIT0047] Trueba S , Isnard S, Barthélémy D, Olson ME. 2016. Trait coordination, mechanical behaviour and growth form plasticity of *Amborella trichopoda* under variation in canopy openness. AoB Plants 8:plw068; doi:10.1093/aobpla/plw068. 27672131 PMC5142121

[CIT0048] Ueno S , Tomaru N, Yoshimaru H, Manabe T, Yamamoto S. 2000. Genetic structure of *Camellia japonica* L. in an old-growth evergreen forest, Tsushima, Japan. Molecular Ecology 9:647–656. 10849281 10.1046/j.1365-294x.2000.00891.x

[CIT0049] Valladares F , Niinemets U. 2007. The architecture of plant crowns: from design rules to light capture and performance. In: Pugnaire FI, Valladares F, eds. Handbook of functional plant ecology, 2nd edn. Boca Raton, FL: CRC Press, 101–149.

[CIT0050] Valladares F , Pearcy RW. 1998. The functional ecology of shoot architecture in sun and shade plants of *Heteromeles arbutifolia* M. Roem., a Californian chaparral shrub. Oecologia 114:1–10. 28307546 10.1007/s004420050413

[CIT0051] Walters MB , Reich RB. 2000. Seed size, nitrogen supply, and growth rate affect tree seedling survival in deep shade. Ecology 81:1887–1901.

[CIT0052] Warton DI , Duursma RA, Falster DS, Taskinen S. 2012. smatr 3—an R package for estimation and inference about allometric lines. Methods in Ecology and Evolution 3:257–259.

[CIT0053] Westoby M , Wright IJ. 2003. The leaf size-twig size spectrum and its relationship to other important spectra of variation among species. Oecologia 135:621–628. 16228258 10.1007/s00442-003-1231-6

[CIT0054] White PS . 1983. Corner’s rules in eastern deciduous trees: allometry and its implications for the adaptive architecture of trees. Bulletin of the Torrey Botanical Club 110:203–212.

[CIT0055] Yang X-D , Yan E-R, Chang SX, Wang X-H, Zhao Y-T, Shi Q-R. 2014. Twig–leaf size relationships in woody plants vary intraspecifically along a soil moisture gradient. Acta Oecologica 60:17–25.

[CIT0056] Zanne AE , Westoby M, Falster DS, Ackerly DD, Loarie SR, Arnold SE, Coomes DA. 2010. Angiosperm wood structure: global patterns in vessel anatomy and their relation to wood density and potential conductivity. American Journal of Botany 97:207–215. 21622380 10.3732/ajb.0900178

