# Supplementary material for: Geographic variation in shoot structure in association with fruit size in an evergreen woody species
Source: AoB Plants. 2021 May 7;13(3):plab023. doi: 10.1093/aobpla/plab023 (PMC8237846; doi:10.1093/aobpla/plab023)
Supplement: plab023_suppl_Supplementary_Materials [file plab023_suppl_supplementary_materials.pdf]

Supporting Information. Data used in the study.

Site	Latitude	FruitVolume	FruitMass	FruitDensity	FruitDiameter	Existence_of_current-year_shoots	Diameter_1yrShoot	Length_1yrShoot	Mass_1yrShoot	LeafNumber_1yrShoot	TotalLeafMass_1yrShoot	MeanLeafMass_1yrShoot	TotalLeafArea_1yrShoot	MeanLeafArea_1yrShoot	LMA	TotalMass_1yrShootComplex	TotalLeafArea_1yrShootComplex
Matsuura	33.41	35.6	50.0	1.40	43.5	No	2.35	8.1	0.50	4	1.13	0.28	66.23	16.56	171.23	17.32	66.23
Matsuura	33.41	59.2	85.4	1.44	53.9	No	2.19	4.2	0.32	5	1.63	0.33	86.15	17.23	189.20	26.63	86.15
Matsuura	33.41	34.7	38.5	1.11	41.4	No	2.59	3.1	0.15	3	0.92	0.31	52.49	17.50	175.47	13.65	52.49
Matsuura	33.41	33.7	39.4	1.17	43.1	No	2.86	7.8	0.35	4	1.07	0.27	79.70	19.92	134.01	14.25	79.70
Matsuura	33.41	21.7	24.9	1.15	36.4	No	2.54	3.4	0.16	4	0.90	0.23	33.32	16.66	270.13	9.76	33.32
Dazaifu	33.54	37.5	44.5	1.19	40.9	Yes	2.56	3.4	0.21	3	1.46	0.49	76.32	25.44	190.65	17.03	137.83
Dazaifu	33.54	40.1	43.5	1.09	43.4	No	2.55	7.9	0.26	5	0.73	0.15	47.32	9.46	153.84	14.94	47.32
Dazaifu	33.54	27.0	30.6	1.13	37.9	Yes	2.99	6.7	0.29	2	0.56	0.28	38.72	19.36	144.37	12.30	117.25
Dazaifu	33.54	21.2	23.8	1.12	36.2	No	2.35	4.4	0.16	2	0.44	0.22	29.78	14.89	147.10	8.97	29.78
Dazaifu	33.54	24.8	27.0	1.09	35.8	No	2.57	3.5	0.32	2	0.40	0.20	NA	NA	NA	10.03	NA
Iizuka	33.68	50.9	52.6	1.03	45.7	Yes	3.34	12.4	0.67	5	1.65	0.33	124.02	24.80	133.04	19.51	173.79
Iizuka	33.68	75.9	90.6	1.19	56.4	No	3.54	17.1	0.20	3	0.61	0.20	62.23	20.74	98.67	26.75	62.23
Iizuka	33.68	37.9	41.3	1.09	43.0	No	3.07	15.3	0.39	5	1.77	0.35	110.45	27.61	160.26	15.51	110.45
Iizuka	33.68	59.9	68.3	1.14	50.9	No	4.68	21.6	0.70	5	2.30	0.46	192.66	38.53	119.59	23.43	192.66
Iizuka	33.68	20.0	22.9	1.15	35.5	No	3.87	25.4	0.46	6	1.59	0.27	118.51	19.75	134.17	10.16	118.51
Hagi	33.46	49.9	62.0	1.24	45.9	Yes	3.51	5.9	0.50	6	1.41	0.24	NA	NA	NA	22.52	NA
Hagi	33.46	58.2	62.0	1.07	47.1	Yes	2.90	3.8	0.28	5	1.14	0.23	63.78	12.76	178.75	20.55	81.11
Hagi	33.46	47.9	69.8	1.46	50.9	No	2.93	12.4	0.45	7	2.15	0.31	136.44	19.49	157.87	23.41	136.44
Hagi	33.46	53.5	55.3	1.03	43.8	Yes	4.13	6.1	0.62	2	0.56	0.28	26.37	13.18	210.87	18.90	64.04
Hagi	33.46	31.4	34.9	1.11	38.6	Yes	3.41	10.1	0.45	2	0.79	0.40	44.79	22.39	176.84	14.24	120.81
Nagasaki	32.58	65.9	68.8	1.04	51.9	Yes	3.33	4.4	0.26	2	0.26	0.13	37.37	18.68	69.05	21.45	47.22
Nagasaki	32.58	75.3	59.5	0.79	45.9	No	3.27	5.4	0.30	1	0.29	0.29	18.68	18.68	155.27	18.77	18.68
Nagasaki	32.58	45.5	48.3	1.06	44.7	No	2.78	4.9	0.24	3	1.09	0.36	75.94	25.31	143.80	16.57	75.94
Nagasaki	32.58	69.9	82.8	1.18	52.0	No	3.27	5.9	0.30	2	0.69	0.35	48.66	24.33	142.02	25.03	48.66
Nagasaki	32.58	85.1	94.1	1.11	54.4	No	3.50	1.7	0.13	1	0.27	0.27	28.97	28.97	92.52	27.18	28.97
Amakusa	32.35	103.9	101.0	0.97	58.0	No	3.44	3.4	0.18	3	0.76	0.25	67.97	22.66	111.08	29.37	67.97
Amakusa	32.35	74.9	100.5	1.34	55.4	Yes	3.83	9.8	0.60	4	2.26	0.57	116.94	29.24	193.43	34.88	279.63
Amakusa	32.35	33.6	45.1	1.34	44.5	Yes	4.02	25.3	1.67	7	2.98	0.43	206.90	29.56	143.98	20.26	266.13
Amakusa	32.35	55.8	54.3	0.97	46.6	No	2.61	2.5	0.11	3	0.91	0.30	51.55	17.18	175.93	17.85	51.55
Amakusa	32.35	204.7	202.6	0.99	69.8	No	4.44	7.9	0.77	4	2.06	0.51	135.17	33.79	152.10	54.05	135.17
Nobeoka	32.58	97.0	122.1	1.26	62.0	Yes	4.56	11.6	0.98	5	3.52	0.70	158.04	31.61	222.60	40.92	320.13
Nobeoka	32.58	46.1	51.4	1.11	45.2	Yes	4.52	13.5	1.13	3	1.80	0.60	100.90	33.63	178.20	21.76	267.34
Nobeoka	32.58	57.5	54.9	0.95	49.1	Yes	3.44	11.7	0.78	2	0.54	0.27	24.16	12.08	223.78	21.49	197.27
Nobeoka	32.58	58.6	50.3	0.86	49.3	No	3.59	20.5	0.91	9	2.42	0.27	145.58	18.20	166.03	19.10	145.58
Owase	34.06	14.2	27.1	1.91	38.7	No	2.28	4.1	0.17	3	0.56	0.19	47.49	15.83	118.13	10.08	47.49
Owase	34.06	4.8	10.4	2.17	23.4	No	2.25	8.1	0.28	5	0.85	0.17	73.44	14.69	115.06	5.28	73.44
Owase	34.06	33.8	32.9	0.97	41.0	No	2.32	5.5	0.15	5	0.99	0.20	97.79	19.56	100.82	12.15	97.79
Owase	34.06	25.9	17.2	0.66	30.9	Yes	3.14	12.3	0.43	2	0.72	0.36	39.47	19.74	182.91	8.87	99.15
Hyakusaiji	35.13	8.7	16.9	1.94	32.0	No	2.44	4.5	0.22	3	1.03	0.34	69.34	23.11	148.53	7.52	69.34
Hyakusaiji	35.13	16.0	27.6	1.73	35.6	No	2.43	6.9	0.25	2	0.78	0.39	75.10	37.55	103.86	10.53	75.10
Hyakusaiji	35.13	21.5	26.4	1.23	36.5	No	2.80	8.3	0.35	2	0.74	0.37	69.42	34.71	105.88	10.23	69.42
Hyakusaiji	35.13	18.8	22.1	1.18	35.1	No	2.12	5.9	0.15	2	0.41	0.21	43.15	21.57	95.49	8.43	43.15
Hyakusaiji	35.13	19.6	23.4	1.19	36.4	No	2.36	2.1	0.14	1	0.09	0.09	7.35	7.35	123.83	8.49	7.35
Koura	35.18	28.6	35.4	1.24	43.1	No	2.27	4.1	0.24	4	1.12	0.28	101.05	25.26	110.84	13.08	101.05
Koura	35.18	8.0	12.7	1.59	34.8	No	2.00	4.5	0.12	3	0.65	0.22	74.55	24.85	86.66	5.69	74.55
Koura	35.18	28.3	16.3	0.58	36.5	No	2.60	2.8	0.12	2	0.59	0.30	59.18	29.59	100.38	6.80	59.18
Koura	35.18	7.4	17.6	2.38	32.7	No	1.84	6.4	0.17	4	0.84	0.21	84.73	21.18	99.62	7.50	84.73
Koura	35.18	32.6	34.9	1.07	39.3	No	2.27	3.7	0.16	2	0.41	0.21	42.85	21.43	96.15	12.15	42.85
Shodo-shima	34.51	20.1	23.1	1.15	32.3	Yes	2.36	7.0	0.19	4	0.68	0.17	44.67	11.17	152.68	9.54	76.57
Shodo-shima	34.51	11.0	16.4	1.49	32.1	No	2.82	9.2	0.42	3	1.17	0.39	64.03	21.34	183.34	7.70	64.03
Shodo-shima	34.52	44.2	45.2	1.02	45.7	No	2.83	11.3	0.44	6	1.51	0.25	100.44	16.74	150.74	16.36	100.44
Shodo-shima	34.52	8.4	13.2	1.57	28.1	No	2.37	3.5	0.13	2	0.34	0.17	30.88	15.44	108.48	5.55	30.88
Shodo-shima	34.52	8.2	11.5	1.40	28.3	No	2.22	1.8	0.08	2	0.17	0.09	16.06	8.03	108.36	4.79	16.06
Kawane-Hommachi	35.14	NA	NA	NA	37.2	Yes	3.27	8.7	0.35	5	2.06	0.41	NA	NA	NA	NA	NA
Kawane-Hommachi	35.14	NA	NA	NA	36.8	Yes	3.27	7.7	0.46	5	1.48	0.30	NA	NA	NA	NA	NA
Kawane-Hommachi	35.14	NA	NA	NA	36.9	Yes	2.74	4.5	0.19	3	1.11	0.37	NA	NA	NA	NA	NA
Kawane-Hommachi	35.14	NA	NA	NA	31.2	Yes	2.60	2.5	0.17	2	0.49	0.24	NA	NA	NA	NA	NA
Kawane-Hommachi	35.14	NA	NA	NA	27.0	No	3.10	6.9	0.33	4	2.46	0.62	120.54	30.13	204.42	NA	NA
Kawane-Hommachi	35.14	NA	NA	NA	34.5	Yes	2.76	8.0	0.33	3	1.31	0.44	NA	NA	NA	NA	NA
